# Railroad Spine

**Published:** 1894-09

**Authors:** John Eric Erichsen

**Affiliations:** 6 Cavendish Place, Cavendish Square W., London


### Correspondence.

### "Railroad Spine."

### LETTER FROM MR. ERICHSEN. *

6 CAVENDISH PLACE, CAVENDISH SQUARE W.,
LONDON, August 7, 1894.

*Dear Doctor Swearingen:*

Let me thank you most cordially for your paper "A Review of Dr. Wallace and the Railroad Surgeons on Spinal Concussion," and for the most generous, able and eloquent manner in which you have, in it, rebutted the calumnious assertions on me made by the persons whose names you give, and whose words you quote.

In no circumstances would I enter into a controversy with persons such as those, who import personal abuse and the imputation of unworthy motives into the discussion of a surgical question. Least of all would I consider it necessary for me to vindicate my character in these respects before the surgical profession of the United States. I can never forget the most hospitable and friendly reception with which I was honored twenty years ago by my surgical brethren of the Union, when I visited many of its principal cities. Ever since that visit it has been my privilege and my pride to count amongst my most valued friends, many of the leading American surgeons.

After more than fifty years of active professional life, I have

* [By the kind permission of Dr. Swearingen, the JOURNAL has the pleaure and satisfaction of giving its readers the following letter from the great English surgeon, John Eric Erichsen, the author of "Railroad Spine." Dr. Swearingen's article in our July number attracted his attention in his secluded English home, and elicited this graceful and grateful acknowledgement. It is published, as will be seen, by Mr. Erichsen's permission, though not written for publication.—EDITOR.]

now sought, in retirement, that repose from my labors to which I consider myself entitled, and I have no intention to allow this to be disturbed by surgical polemics, or by troubling myself by a reply to personal attacks from whatever source they may come. I prefer to leave to friends like yourself the generous task of my defense, should my character and opinions, which for half a century have been subjected to the judgment and criticism of the profession, seem to need their advocacy.

Nearly thirty years have passed since I first brought the subject of railway and other injuries of the nervous system under the notice of the profession. At that time (1866), the pathology of the nervous system and of its injuries was very imperfectly understood, and even its modern nomenclature had not been invented. "Neurosis" and "neurasthenia" even, were unknown terms, and what I then, for want of a better name, called, "concussion of the spine," is now universally recognized and described under the more modern appellation of "traumatic neurasthenia." The morbid states are the same, and the symptoms identical; but the name has been changed, and the modern designation is probably more in accordance with modern views than was the older one. In all my writings on this subject, I have pointed out that the symptoms arising from railway shocks are identical with those that occur from other and more ordinary accidents of civil life, and that these symptoms so occurring had been described by surgeons many years before railways were dreamt of, and fully a century before I had written a line on the subject.

Again thanking you most cordially for your generous sympathy and valuable aid, I am, dear Dr. Swearingen,

Most truly yours,

JOHN ERIC ERICHSEN.

P. S. Pray make any use you like of this letter.

